# Development of a Lateral Flow Strip with a Positive Readout for the On-Site Detection of Aflatoxin B_1_

**DOI:** 10.3390/molecules27154949

**Published:** 2022-08-03

**Authors:** Kemin Shen, Xiaoqin Hu, Linlin Sun, Chun Han, Jianzhou Yang

**Affiliations:** 1 Department of Preventive Medicine, Changzhi Medical College, Changzhi 046000, China; sunlinlin@czmc.edu.cn (L.S.); jzyang@aliyun.com (J.Y.); 2 Department of Chemistry, Changzhi University, Changzhi 046011, China; xiaoqin_hu2021@126.com (X.H.); coldspringfibre@126.com (C.H.)

**Keywords:** positive readout, lateral flow assay, aptamer, aflatoxin B_1_

## Abstract

Aflatoxin B_1_ is one of the contamination indicators for food safety monitoring. The rapid and effective assessment and determination of AFB_1_ in food is of great importance to dietary safety. The lateral flow assay shows advantages in its simplicity, and rapidity, and provides a visual readout, while the available lateral flow assay for AFB_1_ requires a competitive format that produces readings inversely proportional to the AFB_1_ concentration, which is counterintuitive and may lead to a potential misinterpretation of the results. Herein, we developed a positive readout aptamer-based lateral flow strip (Apt-strip) for the detection of AFB_1_. This Apt-strip relies on the competition between AFB_1_ and fluorescein-labeled complementary DNA strands (FAM-cDNA) for affinity binding to limited aptamers against AFB_1_ (AFB_1_-Apt). In the absence of AFB_1_, AFB_1_-Apt hybridizes with FAM-cDNA. No signal at the T-line of the Apt-strip was observed. In contrast, AFB_1_-Apt binds to AFB_1_ in the sample, and then a part of the FAM-cDNA is hybridized with the free AFB_1_-Apt, at which time the other unreacted FAM-cDNA is captured by A35-Apt on the T-line. The signal was observed. This method achieved fast detection of AFB_1_ with a detection limit (DL) of 0.1 ng/mL, positive readout, and increased sensitivity.

## 1. Introduction

Aflatoxin B_1_ (AFB_1_), a fungal metabolite, is highly toxic and carcinogenic to humans and animals [1,2], and it is most commonly found in cereal and oil foods [3]. Long-term exposure to very low levels of AFB_1_ in feed and food is a threat to human and animal health [4,5]. In order to protect the health of humans and animals, many countries and regions have set the maximum allowable limit of AFB_1_ (Table S1) in feed and food [6,7]. The National Food Safety Standards of China (GB 2761-2017) stipulate that AFB_1_ is one of the compulsory inspection items for most foods. Therefore, it is particularly important to establish an accurate and rapid method for the determination of AFB_1_ in food. Currently, liquid-chromatography-based methods, including high-performance liquid chromatography (HPLC) and liquid chromatography–tandem mass spectrometry (LC–MS/MS), are already officially accepted for the quantitative analysis of AFB_1_. However, these methods have the disadvantages of being time consuming, requiring expensive equipment and professional technicians to operate, and are not suitable for the rapid on-site screening of bulk samples. The enzyme-linked immunosorbent assay (ELISA) [8] is an alternative method for the rapid analysis of AFB_1_. Nevertheless, the antibodies are costly and not easily stored, which limit its application in the rapid analysis of AFB_1_.

Lateral flow assay (LFA) has been widely used for the rapid determination of AFB_1_ [9,10,11,12] because of its simplicity, portability, cost effectiveness, and suitability for on-site screening [13,14,15,16]. In 2005, Delmulle et al. prepared an LFA strip using colloidal gold as a signal marker for the rapid assay of AFB_1_ [17]. Subsequently, there has been a significant increase in the number of studies based on colloidal gold LFA for the detection of AFB_1_ [18,19,20]. Until 2014, Wang first reported the use of luminescent nanomaterials as signal amplifiers for the more sensitive detection of AFB_1_ [21]. Since then, various luminescent materials, including quantum dots [22,23,24,25] and fluorescent microspheres [26,27], have been used as signal probes for the LFA technique to enhance the sensitivity of AFB_1_ detection. Recently, in view of the high cost and fallibility of antibodies, aptamer-based lateral flow strips have been developed to detect AFB_1_ [28,29,30]. However, the currently reported LFA methods for detecting AFB_1_ produce readings inversely proportional to the analyte content; i.e., negative samples have the strongest T-line signal intensity, while positive samples have a decreasing T-line signal intensity with an increasing AFB_1_ concentration. This is counterintuitive [31]. In particular, when the AFB_1_ concentration is at the critical value, the sensitivity is low and the observations are not intuitive.

In this paper, we proposed a positive readout aptamer-based lateral flow strip (Apt-strip) for the detection of AFB_1_. The Apt-strip indirectly assays AFB_1_ utilizing the competition of AFB_1_ and 6-FAM labeled DNA complementary strands (FAM-cDNA) to the affinity binding to AFB_1_-Apt. This Apt-strip presents a positive readout. With the increase in AFB_1_ concentration, the greater the amount of FAM-cDNA hybridized at the T-line, and the stronger the fluorescence intensity showed. Through using this Apt-strip, AFB_1_ can be rapidly detected within 15 min, and the detection limit (DL) is less than 0.1 ng/mL. The method possesses the characteristics of good selectivity, a strong anti-interference ability, high sensitivity, and the potential for the rapid and on-site screening of AFB_1_ in the food matrix.

## 2. Results and Discussion

### 2.1. Principle of the Apt-Strip

**Scheme 1:**
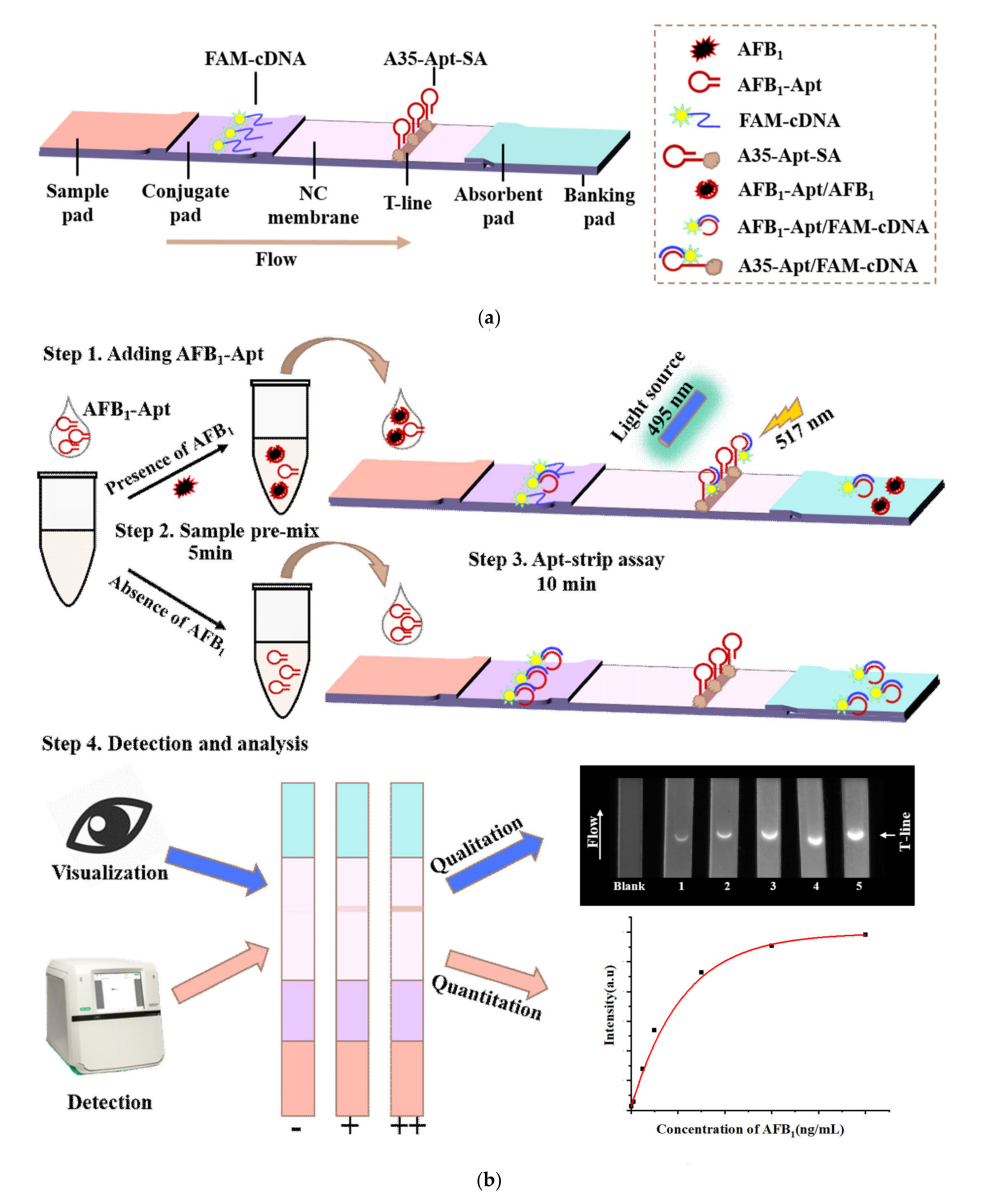
(**a**) Schematic diagram of the Apt-strip analytical device. (**b**) Working principle and detection procedures of the Apt-strip for AFB_1_ detection.

The principle of the Apt-strip is shown in Scheme 1. FAM-cDNA is loaded on the conjugate pad, and the streptavidin-labeled aptamer against AFB_1_ is immobilized at the T-line (Scheme 1a). For AFB_1_-positive samples analysis, AFB_1_ competes with FAM-cDNA to bind to the affinity ligand AFB_1_-Apt in a solution, forming the AFB_1_-Apt/AFB_1_ complex. Then, the AFB_1_-Apt/AFB_1_ complex and free FAM-cDNA probe migrate to the NC membrane, and the A35-Apt of the T-line hybridized with the free FAM-cDNA probe, resulting in the formation of double-stranded DNA (hybridized A35-Apt/FAM-cDNA probe) and the immobilization of FAM-cDNA at the T-line. As a result, a fluorescent spot is observed at the T-line with a ChemiDocTM MP system (Scheme 1b). The fluorescence intensity is increased by increasing the concentration of AFB_1_, which can be used for the quantitative analysis of AFB_1_. For negative samples, the free FAM-cDNA probes hybridize with AFB_1_-Apt in a solution to form double-stranded DNA (AFB_1_-Apt/FAM-cDNA probe) instead of A35-Apt of T-line, leading to a negative fluorescence signal at the T-line.

The results of the qualitative analysis can be identified by the naked eye with the aid of the ChemiDocTM MP system. For quantitative results, this is achieved by further analysis of the image. The specific steps are as follows. First, the strip is imaged using the ChemiDocTM MP system. Then, the fluorescence intensities are converted to numerical values with the help of software Image J. The relationship between the AFB_1_ concentration (X) and fluorescent intensity (Y) can be obtained by constructing a fitting curve.

### 2.2. Optimization of cDNA Length

The affinity of the cDNA and aptamer is a vital factor for the competition of cDNA and AFB_1_ to bind to the aptamer, which differs depending on the length of the cDNA [32,33,34,35,36]. For this reason, we optimized the length of cDNA, ranging from 10 to 16 nucleotides.

**Figure 1:**
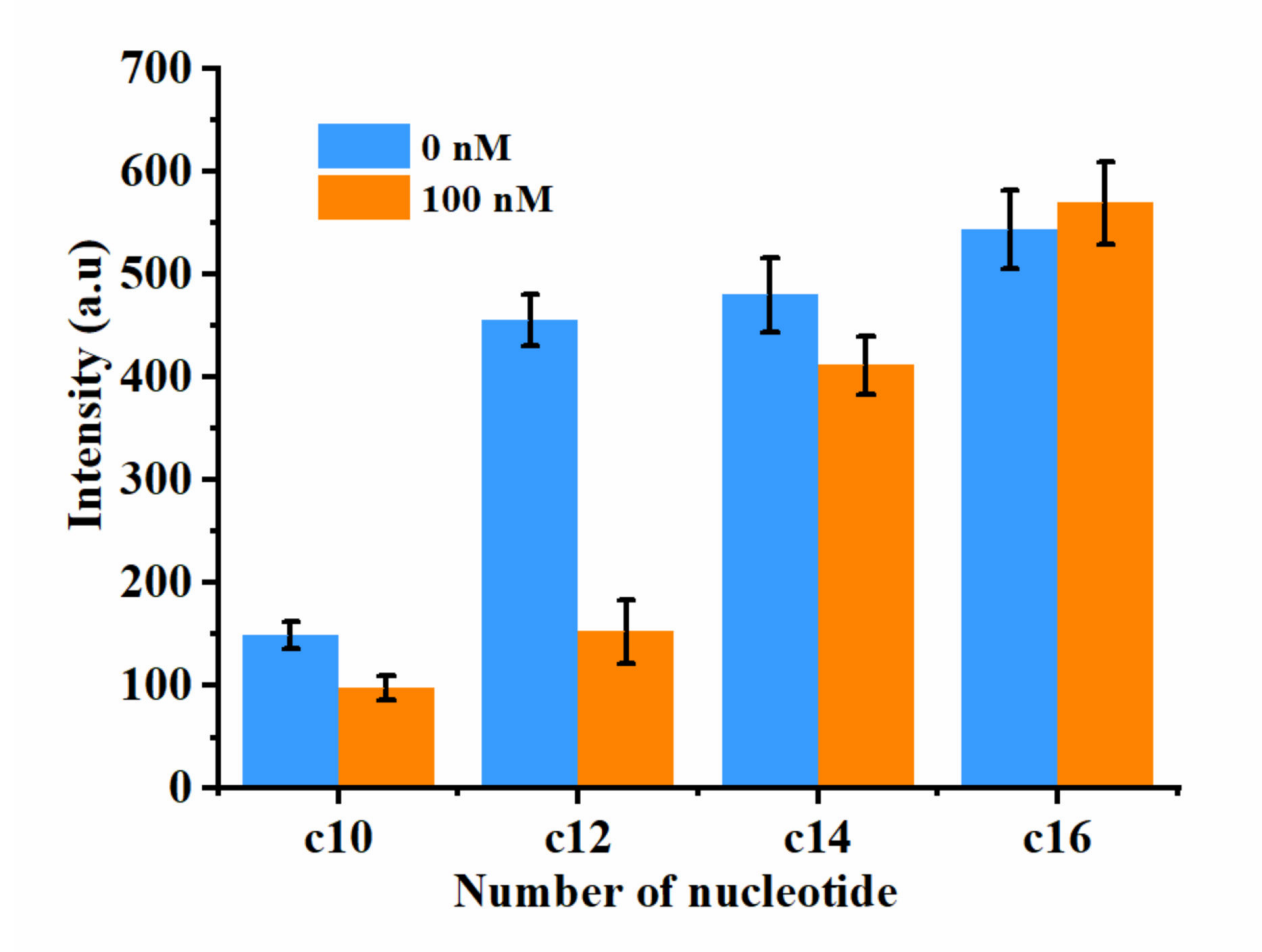
Effects of n-cDNA on the fluorescence intensity of the A35-apt coupled with FAM labeling in the absence (0 nM) or presence (100 nM) of AFB_1_.

As shown in Figure 1, with the increase in cDNA length (n), both blank samples and AFB_1_ samples (100 nM) showed an increasing fluorescence intensity, which resulted from stronger hybridized double-stranded DNA between the cDNA with a longer length and aptamer. The biggest fluorescence intensity change induced by AFB_1_ was obtained when cDNA with 12 nucleotides (12-cDNA). The results indicate that cDNA with more than 12 nucleotides is not in favor of the competition of AFB_1_. Thus, 12-cDNA was selected as the complementary strand for the subsequent tests. 

### 2.3. Analytical Performance of the Apt-Strip

To verify the feasibility of the Apt-strip for the detection of AFB_1_, different concentrations of AFB_1_ (0, 0.1, 1, 5, 10, 30, 60, and 100 ng/mL) were analyzed using the Apt-strip. Buffer C containing10 mM Tris-HCl (pH 7.4), 50 mM NaCl, 10 mM MgCl_2_, and 10% methanol was used as the assay buffer. 

**Figure 2:**
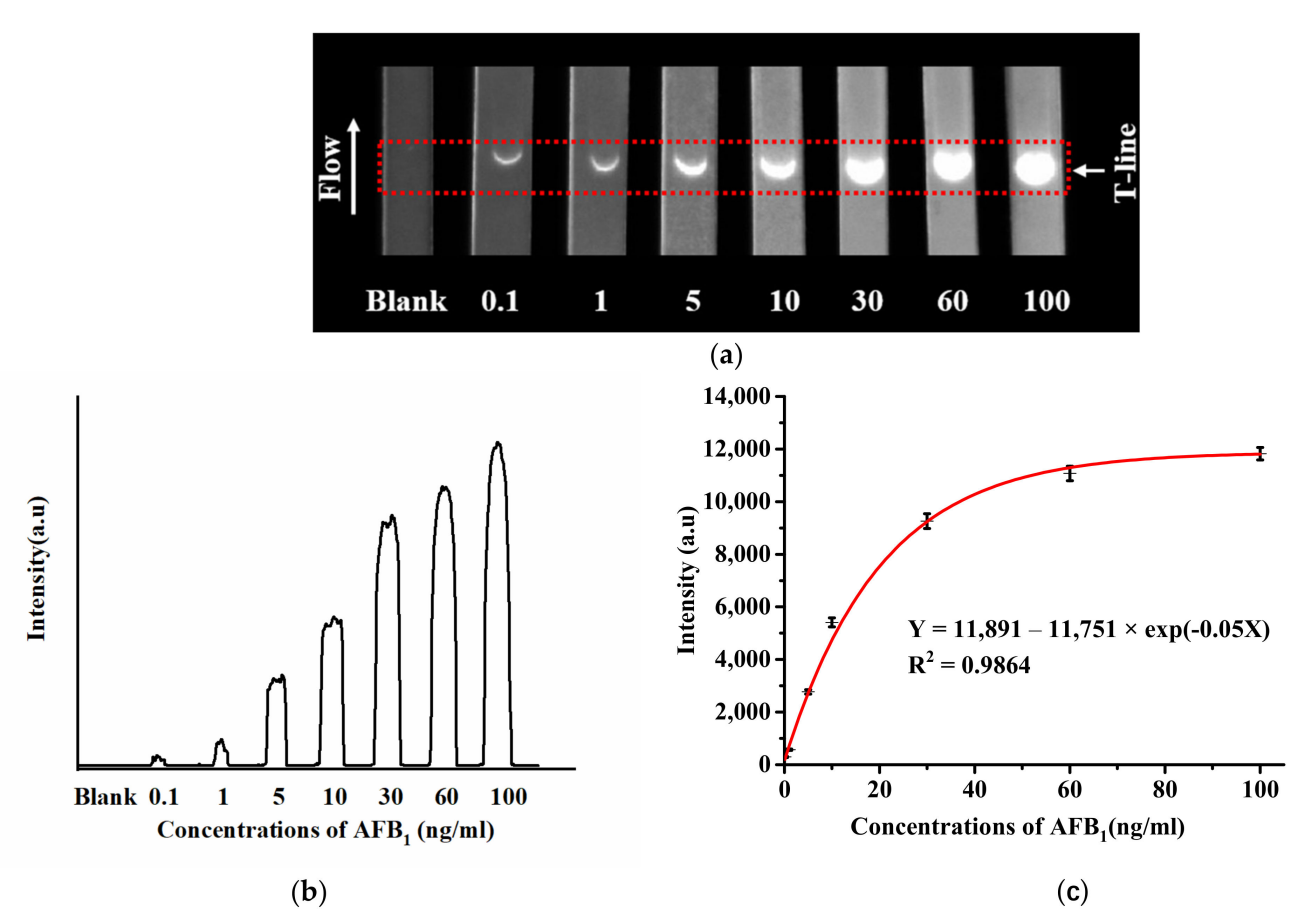
(**a**) Images of Apt-strips after the assay procedures. The numbers below the strips are the standard concentrations of AFB_1_ (ng/mL). (**b**) Fluorescence intensity at the detection line of Apt-strips identified by image J. (**c**) The calibration curve for the quantitation of AFB_1_ using fluorescence intensity versus the concentration of AFB_1_. Error bars are based on three duplicate measurements of different AFB_1_ concentrations.

As shown in Figure 2a, there was no fluorescence signal at the T-line for the blank sample, and the fluorescence signal appeared when the AFB_1_ concentration was 0.1 ng/mL. Then, the fluorescence intensity gradually increased with the increase in AFB_1_ concentration, and the fluorescence intensity reached the highest when the AFB_1_ concentration was 100 ng/mL. The calibration curve (Figure 2c) was constructed using fluorescence intensity (Y) against the concentrations of AFB_1_ (X). The fitted equation was Y = 11,891 − 11,751 × exp (−0.05X) with a reliable correlation coefficient (R^2^ = 0.9864), and the dynamic range of AFB_1_ was from 0.1 ng/mL to 100 ng/mL. The DL for the qualitative evaluation was defined as the minimum concentration when displaying a very weak fluorescence intensity at the T-line, compared with a blank sample [37]. Therefore, the DL was less than 0.1 ng/mL, which was comparable to the value previously reported using other antibody or other aptamer-based LFA (Table 1). In addition, the DL was less than the minimum allowable limit of AFB_1_ (Table S1) set by different countries and regions. Therefore, the Apt-strip could meet the various screening requirements of AFB_1_. It is noteworthy that compared with other strips for AFB_1_ detection, the Apt-strip showed a positive readout, which means the fluorescence signal increased with the increase in AFB_1_ concentration. This positive readout method is more convenient and sensitive for qualitative analysis, especially for the analysis of samples containing very small amounts of AFB_1_. To the best of our knowledge, no AFB_1_ detection method has been reported by positive readout strips, and this strategy is the first report of positive readout test strips for AFB_1_ detection.

To assess the precision and accuracy of the prepared Apt-strip, we chose spiked samples of corn and wheat as the food matrixes. All of the food samples were first analyzed by the HPLC-FLD method to ensure there was no contamination of AFB_1_.

First, we accurately weighed 5 g of the sample into a 50 mL PP tube. Then, we added the appropriate amount of different concentrations of the AFB_1_ standard solution. The spiked samples were processed as in Section 3.6. The extracts were analyzed using the Apt-strip. Experimental results in Table 2 indicate that the recoveries were in the range of 50.0–97.0% for AFB_1_ with relative standard deviations (RSDs) less than 36.7%, and were acceptable within the requirements of No. 401/2006 [7]. These results suggest that the Apt-strip developed in this study can be used for the quantitative and qualitative detection of AFB_1_ in real samples.

**Figure 3:**
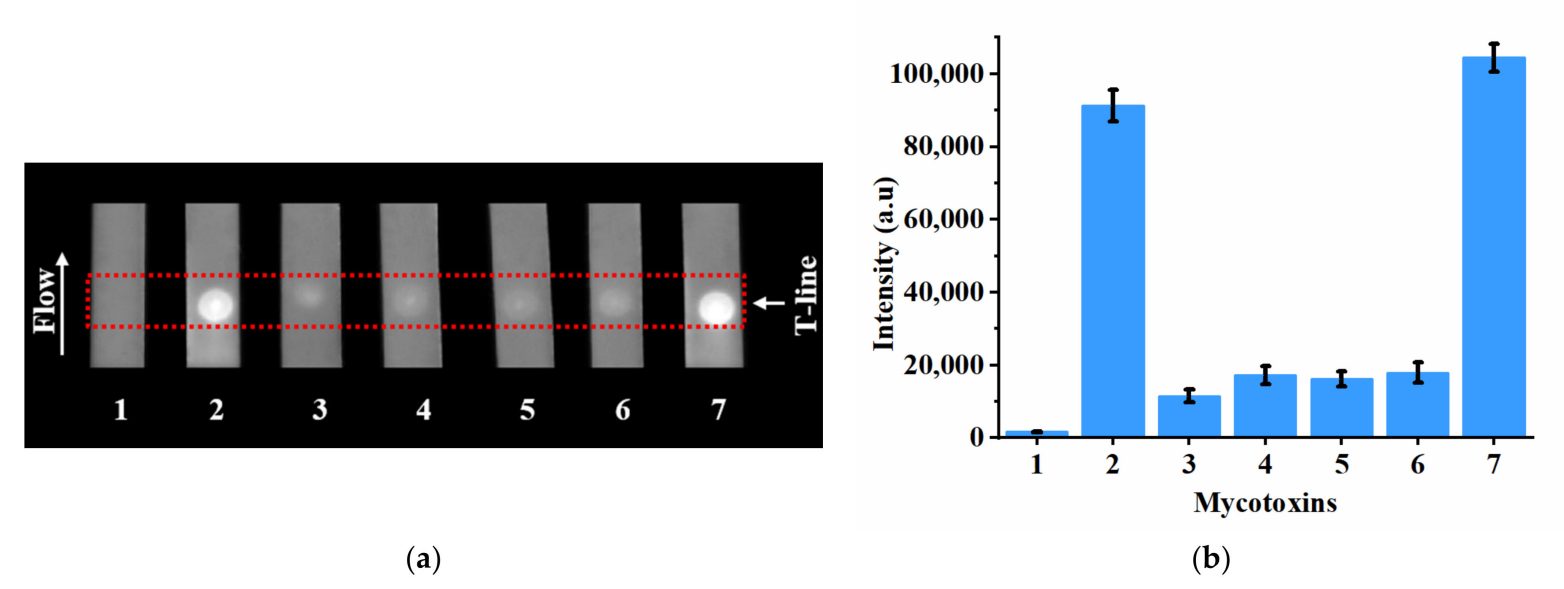
Specificity verification of the Apt-strip by comparing AFB_1_ (50 ng/mL) and four other mycotoxins (50 ng/mL). (**a**) Images of the results of the Apt-strip assaying various toxins. (**b**) Comparison of intensity of various toxins detected using Apt-strip. From left to right: (**1**) blank; (**2**) AFB_1_; (**3**) OTA; (**4**) AFG_1_; (**5**) AFG_2_; (**6**) ZEN; (**7**) mixture of AFB_1_ and other four mycotoxins.

To verify the specificity of the Apt-strip for AFB_1_, we tested several mycotoxins, including OTA, AFG_1_, AFG_2_, ZAE, and the mixture of AFB_1_ with the above mycotoxins together. AFB_1_ and other mycotoxins were all tested at 50 ng/mL. The results are shown in Figure 3. The tested mycotoxins did not cause a significant increase in intensity (Figure 3b), while AFB_1_ induced a clear fluorescence spot (Figure 3a). The mixture of these mycotoxins with AFB_1_ presented a similar fluorescence spot as for the AFB_1_ sample. The results indicate that the Apt-strip had a good selectivity for AFB_1_ detection.

**Figure 4:**
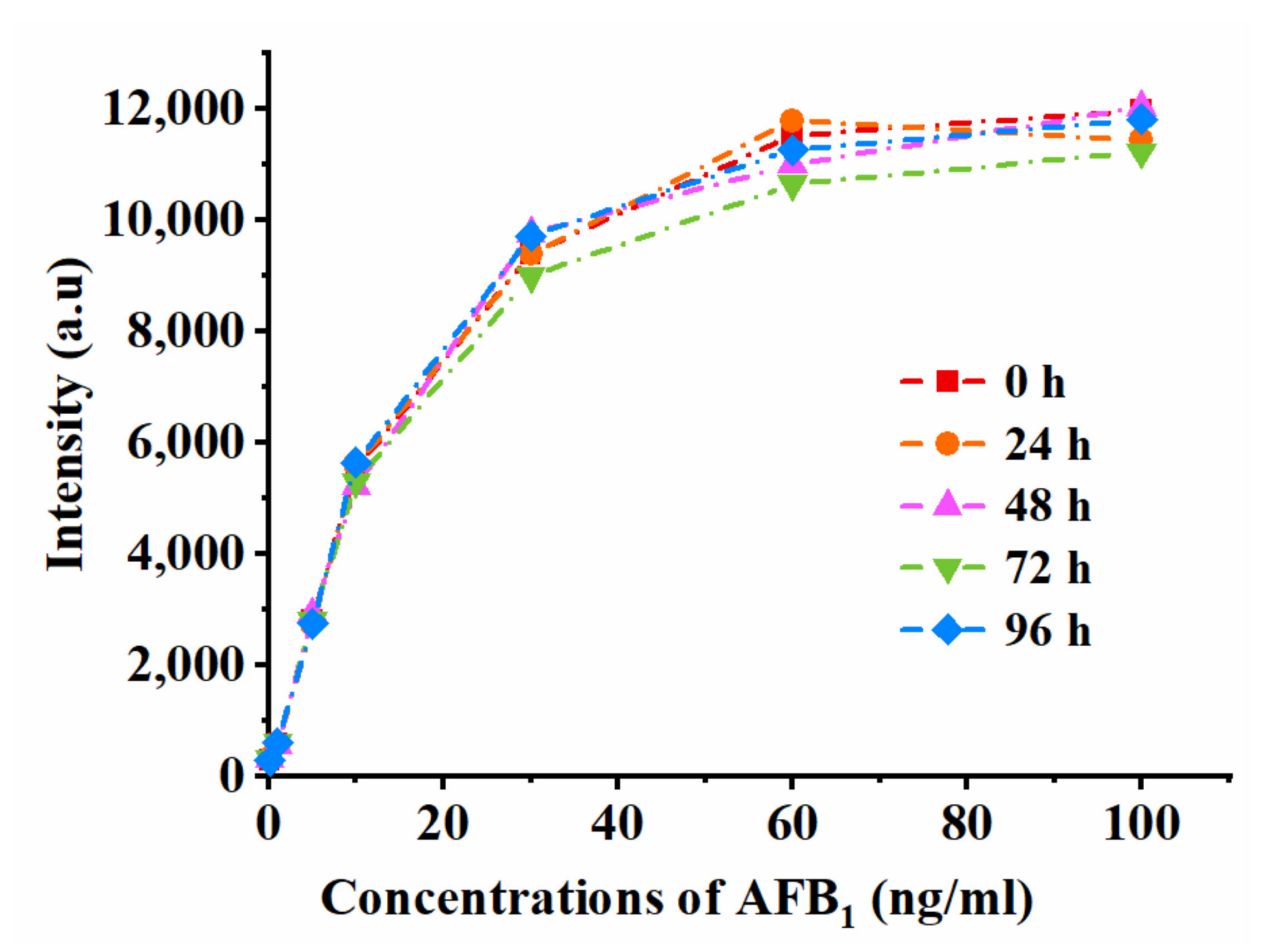
Trend plots of the response values of the Apt-strip to different concentrations of AFB_1_ with a 24 h interval.

To evaluate the stability of the Apt-strip, stability experiments over time were carried out. Apt-strips of the same batch were placed in foil pouches with a desiccant, and were stored at room temperature for 90 days. Then, the strips were used to detect different concentrations of AFB_1_ (0, 0.1, 1, 5, 10, 30, 60, and 100 ng/mL), and the detection was performed once every 24 h. The Apt-strip assayed different concentrations of AFB_1_, and the RSDs were all less than 5.3% (Table 3), indicating that the repeatability of the Apt-strip remained consistent. Trend plots with a 24 h interval (Figure 4) show that there was no change in trend over time. These results show that the performance of the Apt-strip remained stable after 90 days of storage at room temperature.

### 2.4. Detection of AFB_1_ in Real Samples

**Figure 5:**
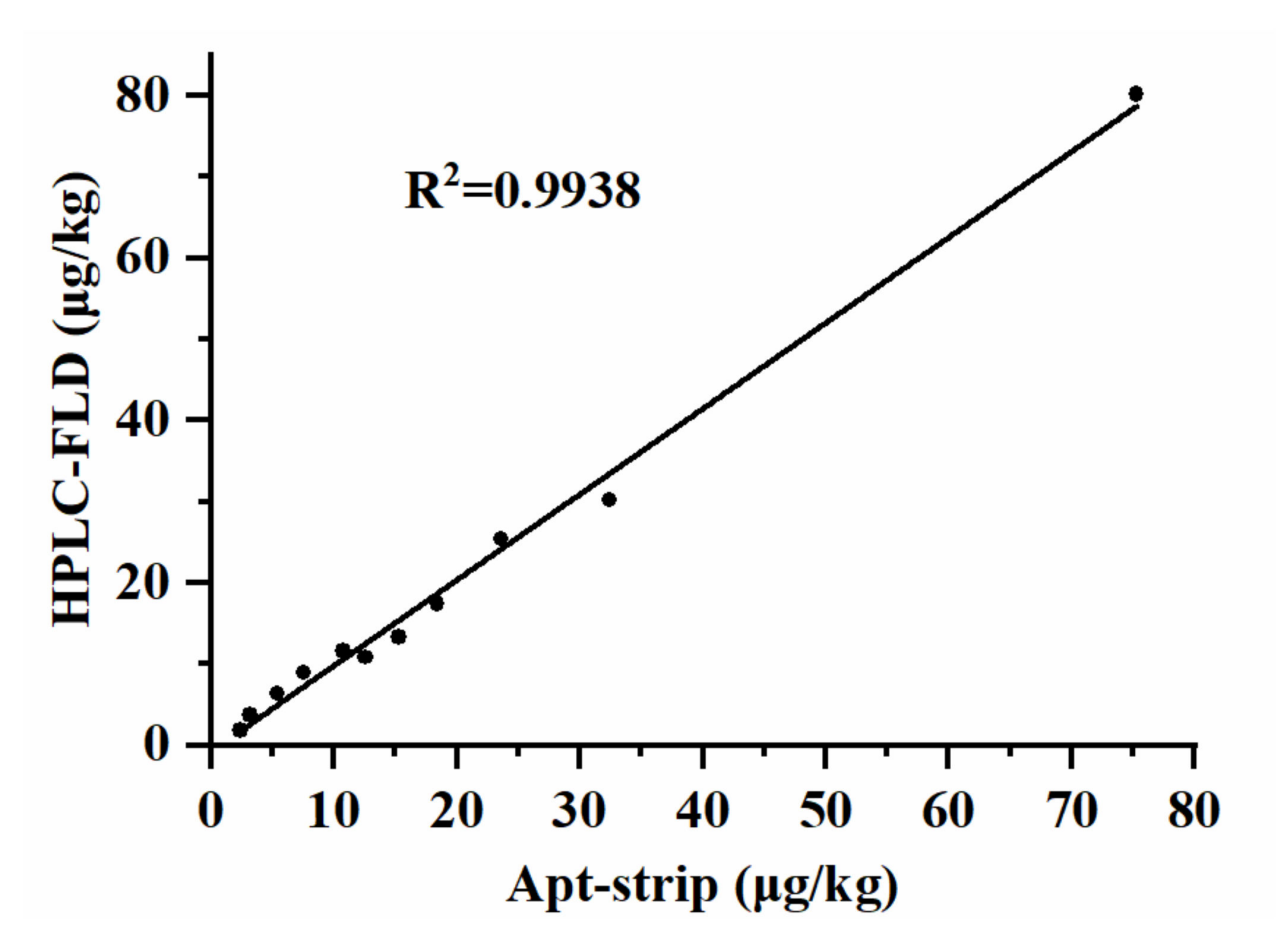
Correlation between Apt-strip and HPLC-FLD for the quantification of AFB_1_ in real samples (*n* = 3).

To evaluate the practicability of the Apt-strip in real samples, 25 batches real samples were analyzed using the Apt-strip and HPLC-FLD. First, 25 batches of real samples, composed of 13 corn, 6 wheat, and 6 sorghum, were collected from Changzhi City, Shanxi Province, China. Table 4 shows that 11 out of 25 samples were found to have AFB_1_. The residual level of AFB_1_ ranged from 2.4 ± 0.7 µg/kg to 75.3 ± 5.3 µg/kg. All the samples were confirmed by HPLC-FLD analysis. The results of the Apt-strip were not false-positive or false-negative. Figure 5 indicates that the two methods yielded consistent results with a good correlation (R^2^ = 0.9938). The above results demonstrate that the Apt-strip is reliable and accurate for thepractical qualitative and quantitative detection of AFB_1_ in real samples, and is a portable tool for the on-site detection of AFB_1_.

## 3. Materials and Methods

### 3.1. Reagents and Materials

AFB_1_, aflatoxin G_1_ (AFG_1_), aflatoxin G_2_ (AFG_2_), zearalenone (ZEN), and ochratoxin A (OTA) were purchased from Aladdin (Shanghai) Co., Ltd. (Shanghai, China). First, 96-Microwell plates (flat bottom) were purchased from Thermo Fisher Scientific Inc. (Shanghai, China). The nitrocellulose (NC) membrane, sample pad, conjugate pad, absorbent pad, and PVC backing were purchased from Shanghai Jieyi Biotechnology Co., Ltd. (Shanghai, China). All of the other reagents were purchased from Sangon Biotech (Shanghai) Co., Ltd. (Shanghai, China). 

### 3.2. Aptamer and DNA Probes

The aptamer sequence was referenced from our previously published paper [44]. The complementary DNA (cDNA) of the aptamer was designed for the development of LFA. Aptamer and cDNA were ordered from Sangon Biotechnology Co., Ltd. (Shanghai, China), and the sequences are listed in Table 5. A35 had one biotin label at the 3’ terminus and a TEG (triethylene glycol) linker. The cDNA each had one 6-FAM label at the 3’ terminus.

### 3.3. Preparation of A35-Apt Coated Microplates

The A35-Apt was coated on the surface of 96-well black microplates by the following steps. Firstly, 100 μL of streptavidin (SA, 10 μg/mL) in 0.1 M Na_2_CO_3_ solution (pH 9.6) was added into the wells of the microplates and they were incubated overnight at 4 °C. Then, after washing three times with 150 μL of buffer A (10 mM Tris-HCl (pH 7.5), 150 mM NaCl, and 0.1% Tween 20), the wells of the microplate were bound with 200 μL of buffer A containing 10 mg/mL BSA at 25 °C for 2 h under shaking. The wells were washed with 250 μL of buffer A. Next, 100 μL of buffer A containing 25 nM biotinylated A35-Apt was added to the SA coated wells, and the mixture was incubated for 1 h at 25 °C under shaking. Finally, the wells were washed three times with 200 μL of buffer B (10 mM Tris-HCl (pH 7.5), 10 mM MgCl_2_, and 50 mM NaCl), and the A35-Apt coated microplate was ready for the analysis of targets.

### 3.4. Optimization Procedure of cDNA Length

The optimization was as follows. Firstly, 100 μL of buffer B containing 100 nM AFB_1_ was added to each of the four wells of the A35-Apt coated microplates, and 100 μL of 20 nM n-cDNA (*n* = 10, 12, 14, and 16) was added sequentially. Secondly, the mixtures were incubated for 10 min at room temperature, and the wells were washed three times with buffer B. Thirdly, 100 μL of buffer B was added sequentially to the four wells, and the fluorescence intensity was measured using a multifunctional enzyme marker (Infinite M Plex, λ_ex/em_ = 495/517 nm). Meanwhile, buffer B without AFB_1_ (AFB_1_ 0 nM) was used as a control.

### 3.5. Manufacture of the Apt-Strip

The structure of the Apt-strip is shown in Scheme 1a. Firstly, an NC membrane was treated with a streptavidin labeled aptamer against AFB_1_ (A35-Apt-SA, 30 μM, 0.5 μL/cm) for T-line, and a conjugate pad was treated with cDNA (2 μM, 5 μL/cm). Then, the treated NC membrane and conjugate pad were dried at 37 °C for 20 min. Secondly, the test strips were assembled according to Scheme 1a, and the joints overlapped by 2 mm. Thirdly, the strip was cut into 4 mm wide test strips and stored in a desiccator at room temperature until use. In our experiments, we omitted the preparation of the control line (C-line) because it is very simple and always effective in actual operation [43]. Note that the final Apt-strip design would include a control line.

### 3.6. Assay Procedure of AFB_1_ in Food Samples

The food samples were prepared according to the procedure described in GB 5009.22-2016 of China. Briefly, solid samples of food were ground, weighed (5 g), and transferred into a 50 mL PP tube, and then extracted with 20 mL methanol/water (70:30, *v*/*v*) by homogenizing for 5 min, followed by centrifugation at 5000 r/min for 5 min at 4 °C. The supernatant was collected and diluted seven times with buffer B to obtain a sample solution. 

The procedure for determining AFB_1_ using Apt-strip is as follows. Forst, 2 μL of AFB_1_-Apt (2 μM) and 50 μL of the sample solution were mixed in a PP tube and incubated at 25 ° C for 5 min, and then the mixture was placed on the Apt-strip. After 10 min, the results were observed by the ChemiDocTM MP system. Then, the fluorescence intensity at the T-line was scanned with Image J software to achieve a quantitative analysis of the assay results. All of the assays were repeated three times.

### 3.7. HPLC-FLD Confirmation

The reliability and practicability of the Apt-strip was further confirmed by HPLC-FLD analysis using the retention time and chromatographic peak area of AFB_1_ as the basic parameters. The specific analytical conditions are listed in the supporting information (HPLC-FLD conditions).

## 4. Conclusions

In this paper, we report a positive readout Apt-strip for the detection of AFB_1_ in food. We used competition between AFB_1_ and FAM-cDNA to bind the limited AFB_1_-Apt and free FAM-cDNA hybridized to A35-Apt at the T-line, and detected AFB_1_ indirectly by measuring the fluorescence intensity at the T-line. The DL of this method was 0.1 ng/mL for AFB_1_ in buffer and 0.3 ng/g in food, with a dynamic range of 0.1–100 ng/mL in the buffer and an R^2^ of 0.9864. The sensitivity and selectivity were very satisfactory. The validation results on the spiked samples and real samples show that the method is dependable. In addition, the Apt-strips are low-cost, and the detection process does not require specialized technicians and it can be used as a rapid scanning tool for food industries or regulatory laboratories. This work is expected to provide new insight into the detection of other food contaminants.

## Figures and Tables

**Table 1:** Comparison of the Apt-strip in this study with other methods for AFB_1_ detection based on LFA.

Type	T-line	Probes	Signal Readout	DL	Signal Reader	Ref.
Antibody-strip	AFB_1_-BSA	colloidal gold-mAb	negative	5 μg/kg	Strip reader	[5]
Antibody-strip	AFB_1_-BSA	QB-mAbs	negative	1 ng/mL	A fluorescent reader	[23]
Antibody-strip	AFB_1_-BSA	mAb@Eu- nanosphere	negative	0.16 μg/kg	Fluorescent strip reader	[38]
Antibody-strip	AFB_1_-BSA	Ab-GNPs	negative	0.1 μg/kg	Strip reader	[39]
Antibody-strip	AFB_1_–OVA	gold-labeled antibody	negative	5 μg/kg	ICheck-III card reader	[10]
Nanozyme-strip	AFB_1_-BSA	MnO_2_NSs-mAb	negative	15 pg/mL	Smart phone	[40]
Aptamer-strip	AFB_1_-BSA	Cy5-Aptamer	negative	0.1 μg/kg	Fluorescent strip reader	[41]
Aptamer-strip	DNA single strand	Cy5-Aptamer	negative	0.16 μg/kg	The portable multi-target reader	[28]
Aptamer-strip	SA	Cy5-Aptamer	negative	0.1 ng/mL	ChemiDocTM MP System	[42]
Aptamer-strip	bio-DNA probe-SA	NGPs-Aptamer	negative	0.5 μg/mL	Strip reader	[43]
				5 μg/mL	Naked eye	
Aptamer-strip	DNA single strand	FAM-Aptamer	Positive	<0.1 ng/mL	ChemiDocTM MP System	This work

**Table 2:** Recovery results of spiked samples using the Apt-strip.

Sample	AFB_1_ spiked (ng/g)	Detected (ng/g)	Recovery (%)	RSD (%)
Corn	1	0.5 ^a^	50.0	36.7
	3	2.9	96.7	8.7
	10	9.7	97.0	6.1
Wheat	1	0.5	50.0	21.7
	3	2.5	83.3	8.4
	10	9.6	96.0	7.3

^a^ Take three parallel samples. Each sample was measured three times, and the average value was used for data processing.

**Table 3:** Stability results of the different concentrations of AFB_1_ using Apt-strip (*n* = 5).

Concentrations of AFB_1_ (ng/g)	Intensity (a.u) ^a^	RSD (%)
0.1	294.7 ± 15.4	5.3
1	582.9 ± 21.7	3.8
5	2800.7 ± 75.9	2.8
10	5460.2 ± 188.2	3.5
30	9457.6 ± 312.8	3.4
60	11,249.7 ± 444.8	4.0
100	11,700.2 ± 352.2	3.1

^a^ Mean ± SD, is the mean and standard deviation of five measurements with a 24 h interval between each test.

**Table 4:** Analysis results of the Apt-strip and HPLC-FLD for AFB_1_ in maize, wheat, and sorghum samples (*n* = 3).

Category	Sample No	Apt-Strip ^a^ (μg/kg)	HPLC-FLD (μg/kg)
Maize	1	ND ^b^	ND
	2	ND	ND
	3	5.4 ± 1.2	6.3 ± 0.8
	4	ND	ND
	5	12.6 ± 1.8	10.8 ± 0.8
	6	32.4 ± 2.4	30.2 ± 1.0
	7	ND	0.1
	8	75.3 ± 5.3	80.2 ± 2.4
	9	18.4 ± 1.8	17.4 ± 0.9
	10	10.8 ± 0.9	11.6 ± 0.6
	11	ND	ND
	12	7.6 ± 1.6	8.9 ± 0.6
	13	ND	ND
Wheat	14	ND	ND
	15	ND	ND
	16	3.2 ± 1.5	3.7 ± 1.1
	17	23.6 ± 3.4	25.4 ± 0.7
	18	ND	ND
	19	ND	ND
Sorghum	20	ND	ND
	21	ND	ND
	22	15.3 ± 1.9	13.3 ± 0.6
	23	2.4 ± 0.7	1.8 ± 1.2
	24	ND	ND
	25	ND	ND

^a^ Values are expressed as the mean ± standard deviation. ^b^ ND: None detected (<DL).

**Table 5:** Sequences of aptamer and cDNA.

Name	Sequences (5’-3’)
A35-Apt	TGCACGTGTTGTCTCTCTGTGTCTCGTGCTTTTTT-biotin-TEG
AFB_1_-Apt	TGCACGTGTTGTCTCTCTGTGTCTCGTGC
10-cDNA	AACACGTGCA-6-FAM
12-cDNA	ACAACACGTGCA-6-FAM
14-cDNA	AGACAACACGTGCA-6-FAM
16-cDNA	AGAGACAACACGTGCA-6-FAM

## Data Availability

Not applicable.

## Supplementary Materials

The following supporting information can be downloaded at: https://www.mdpi.com/article/10.3390/molecules27154949/s1, Table S1: The maximum tolerable limit of AFB_1_ in feed and food set by different countries; HPLC-FLD conditions.
