# Supplementary material for: Development of a Lateral Flow Strip with a Positive Readout for the On-Site Detection of Aflatoxin B1
Source: Molecules. 2022 Aug 3;27(15):4949. doi: 10.3390/molecules27154949 (PMC9370625; doi:10.3390/molecules27154949)
Supplement: Supplementary file 1 [file molecules-27-04949-s001.zip › molecules-1810460-supplementary.pdf]

Supplementary Materials

Development of a Lateral Flow Strip with a Positive Readout for the On-Site Detection of Aflatoxin B1

Kemin Shen [1,*,†], Xiaoqin Hu [2,†], Linlin Sun [1], Chun Han [2] and Jianzhou Yang [1]

1 Department of Preventive Medicine, Changzhi Medical College, Changzhi 046000, China; shenkm@czmc.edu.cn (K.S.); sunlinlin@czmc.edu.cn (L.S.); jzyang@aliyun.com (J.Y.)

2 Department of Chemistry, Changzhi University, Changzhi 046011, China; xiaoqin_hu2021@126.com (X.H.); coldspringfibre@126.com (C.H.)

* Correspondence: shenkm@czmc.edu.cn; Tel: +86-355-3151068

† Those authors contributed equally to this work.

Table S1 The maximum tolerable limit of AFB_1 in feed and food set by different countries and regions.

Category	Organization	Products	Maximum tolerable limit (μg/kg)
Food	China	Corn and its products	20
	European Union	Corn in food ingredients	5.0
	South Africa	all foods	5.0
Feedstuffs	China	Corn (meal)	50
		Concentrate Supplement for Beef Cattle	50
		Compound feed for growing chickens	20
		Compound feed for growing pigs	20
	European Union	All feed ingredients	20
		compound feed for pigs	20
		Other compound feed	10
		Compound feed for dairy animals	5

HPLC-FLD conditions

HPLC separation was tried on the following: C18 (ODS, 250 mm × 4.6 mm, 5 μm, MZ Analysentechnik). A ternary gradient comprising of A (water) and B (acetonitrile : methanol=50:50,V/V) at a flow rate of 1.0 mL/min was used as mobile phase. The gradient profile was as follows: 0 min, 76% A, 24% B; 6 min, 76% A, 24% B; 8 min, 65% A, 35% B; 10 min, 65% A, 35% B; 10.2 min, 100% B; 11.2 min, 100%; 11.5 min, 76% A, 24% B; and 20 min, 76% A, 24% B. The column temperature was 40 ℃ and the excitation and emission wavelengths were 495 nm and 517 nm. Quantification was carried out by use of external calibration curves.
